# Corona crimes: How pandemic narratives change criminal landscapes

**DOI:** 10.1177/1362480620981637

**Published:** 2022-05

**Authors:** Sveinung Sandberg, Gustavo Fondevila

**Affiliations:** University of Oslo (UiO), Norway; Center for Economic Research and Teaching (CIDE), Mexico

**Keywords:** Covid-19, epidemic psychology, Latin America, Mexico, narrative criminology, pandemic

## Abstract

The epidemic psychology of pandemics creates an atmosphere of panic and fear that can expedite new laws and facilitate criminogenic narrative arousal. Using narrative criminology, we discuss crimes that emerged from pandemic narratives in the early phases of the disease in Mexico. We show how pandemic master narratives have unexpected criminogenic effects; can be negotiated to make them criminogenic; and are opposed by more fundamentally criminogenic counter-narratives. We also show how pandemics repurpose justifications for traditional crimes and offer an opportunity for narrative repositioning of “criminals”. Societal crises intensify the continuous narrative negotiation that always underlies the meaning of crime. Pandemics can therefore act as a prism through which social scientists can see how crime is an ongoing narrative accomplishment.

## Introduction

The fundamental criminological question “what is crime?” becomes even more urgent in pandemics. While crime is always the outcome of a particular societal and historical context, and is constantly under negotiation, this is particularly evident during crises and great societal change. Such times also give rise to new types of crime, reignite old ones and repurpose justifications for crime. The meaning and general understandings of laws and crimes that emerge during pandemics are less fixed than they are for longer-standing ones, and leave more room for narrative inventiveness, negotiation and resistance. Christie (2004) famously stated that there is no such thing as “crime”. With this seemingly provocative statement, he perhaps only pointed out the obvious: namely, that what is defined as crime is always in flux, and reliant upon state definitions and popular perceptions. Understandings of crime are in constant negotiation with involved institutional stakeholders, mass media, politics and public opinion.

Christie’s statement recalls developments within critical social studies, the turn to language, and constructivism in one simple sentence. In criminology, such views have been expressed by critical criminology (Michalowski, 1996), constitutive criminology (Henry and Milovanovic, 1996), cultural criminology (Ferrell et al., 2008) and narrative criminology (Presser and Sandberg, 2015). In different ways, these perspectives study how crime and harm emerge from, and are embedded in language, and have pointed out that what is considered “criminal” at any particular moment in history is always defined by those in power. Moreover, what is punishable and how it is punished frequently changes, and certain behaviors are sometimes described as “criminal” even when they may not be against the law, while other acts may be acceptable in public opinion even though they are against the law. Crime should thus be seen as an ongoing accomplishment, and a process where state institutions and public opinion come to a temporary agreement on what should be understood as criminal and how it should be sanctioned. Catastrophes, crises and other great societal changes increase and intensify these processes (Strong, 1990), working as catalysts for changes in both criminal behavior and narrative interpretation. During a state of perceived emergency, there is more at stake, and thus sometimes a greater narrative openness (Punday, 2012) or narrative ambiguity (Polletta, 2006) in terms of how behavior should be interpreted. There is also a greater acceptance of radical changes.

The Covid-19 pandemic changed established patterns in traditional crime (e.g. Ashby, 2020; Boserup et al., 2020; Shayegh and Malpede, 2020). During the early phases of the coronavirus pandemic, new forms of criminal behavior also emerged and older ones were reignited. In Latin America, some communities began to illegally block roads in order to prevent the spread of the disease, there were attacks on healthcare workers out of fear of infection and looting and other crimes occurred, justified by the unfolding crisis. The coronavirus pandemic resulted in the introduction of new laws limiting social interaction and mobility, and thus also, in new possible crimes such as breaking curfews, not wearing masks in public or breaking other government or state regulations. In some countries in the region, criminal organizations also started distributing food to help vulnerable communities, sanitizing public transport and preventing people from leaving their homes by enforcing the quarantine. Where the State took no responsibility for these actions, drug cartels and gangs attempted to change their public image by assuming the role of the State and benevolent organizations.

In this study, we explore the changes that occurred in the criminal landscape in Mexico during the first phases of the coronavirus pandemic. We argue that such changes should be seen in light of pandemic master narratives and the negotiation with and opposition to these dominant understandings of the crisis. Using insights from epidemic psychology (Strong, 1990) and narrative criminology (Fleetwood et al., 2019), we explore how changes in the criminal landscape were linked to pandemic narratives. Arguably, insights gained from this Latin American case study can contribute to a better theoretical understanding of crime, as well as to studies of law violations during societal crises, pandemics and great social change.

## Narrative criminology and pandemic stories

Narrative criminology began with Presser’s (2009) emphasis on how stories should be seen as *constitutive* of crime and harm. Researchers in this tradition have primarily studied the narratives of offenders (Fleetwood et al., 2019; Presser and Sandberg, 2015; Sandberg and Ugelvik, 2016), although increasingly of victims as well (e.g. Cook and Walklate, 2019; Hourigan, 2019). Studies of how the State or state agents instigate harm also form part of this approach (see Dollinger and Heppchen, 2019; Keeton, 2015; Kurtz and Colburn, 2019; Offit, 2019; Petintseva, 2019; Ugelvik, 2016). For a fundamentally social constructivist approach, however, there has been surprisingly little emphasis on the societal construction of crime. Narrative criminologists have taken for granted, rather than studied, that understandings of “crime” and “criminal” are narrative accomplishments within a particular historical and societal setting. The main exceptions are Tognato’s (2015) study of the changing perceptions of tax evasion in Italy and some of Presser’s (2013, 2018) studies of mass harm. Thus far, narrative criminology has not placed any real emphasis on changes in formal state definitions and popular public understandings of crime.

Summarizing narrative theory, Presser (2018) describes how narratives feature temporality (at least two events connected with each other in time), causality (one event leading to another), action, conflict, transformation, meaning, situatedness and things unsaid. Stories also have characters (Propp, 1968), with heroes and villains arguably the most important ones. Following the defining characteristic of narrative criminology, Presser (2018: 57, emphasis in original) claims that narratives “set out an *integrated common sense of action*”, and emphasizes that some stories arouse dramatic or violent action (Presser, 2018). The arousal that may accompany stories is crucial to understanding the possible criminogenic effects of narratives. Stories drive people to do things, not at all mechanically (in the same way, for everyone, always) as assumed by one critic of narrative criminology (Laws, 2020), but by providing ideas or scripts of what is possible and creating the environment, including the affective atmosphere, that function as a call to action.

Apocalyptic narratives are particularly arousing. They come with “intense character polarization”, “the highest and lowest of human emotions” and are especially effective “at generating and legitimating massive society-wide sacrifice” (Smith, 2005: 26–27). By creating an atmosphere of catastrophe, sometimes claiming that the world as we know it is coming to an end, and pitting ultimate good against ultimate evil, these stories call for heroic action, such as terrorism, rebellion, uprisings and warfare (e.g. McCants, 2015; Mason, 2002; Smith, 2005). Apocalyptic ideation imbues life with a telos, with a sense of urgency, and this urgency in turn compels people to consider a broader range of actions than they would have done under other circumstances. The crisis creates a narrative environment where what would otherwise have been considered radical, unthinkable or impossible, can be accepted or even seem unavoidable. Apocalyptic narratives have a Manichean logic (actors are evil or good, arguments are black or white) that makes them particularly fit for mobilization; sometimes fostering “narratives of rebellion” where the “prospect of danger and death are central to the dramatic logic” that drives action (Joosse, 2019: 5).

Epidemic psychology was developed based on studies of societal responses to AIDS, and has received renewed scholarly attention with the Covid-19 pandemic (e.g. Monaghan, 2020). Strong (1990: 249) describes how epidemics can be an “extraordinary emotional maelstrom” triggering “plagues of fear, panic, suspicion and stigma”. This psycho-social form has both collective and individual impact. Epidemic psychology feeds on apocalyptic narratives and has the potential to affect almost anyone in society (because anyone can be infected). Medical epidemics are thus often followed by epidemics of suspicion, fear and stigmatization (Strong, 1990). In this extraordinary state new “moral entrepreneurs” (Becker, 1963) may appear, trying to take advantage of the havoc. Importantly, epidemic psychology is an ideal type, seldom seen in its strongest form, but often seen “in its purest shape when a disease is new, unexpected, or particularly devastating” (Strong, 1990: 250). The first months of the Covid-19 pandemic are emphasized in this article.

The pandemic master narratives, introduced and sponsored by governments and mass media at the start of the coronavirus crisis, sometimes had characteristics of apocalyptic narratives. Stories including estimations of possible deaths, detailed descriptions of possible scenarios at overcrowded hospitals, the threat of social unrest and uncertainty regarding who was at risk, all came together to create an epidemic psychology that justified massive, society-wide sacrifice. The apocalyptic narrative tone provides pandemic stories with great power and explains their potential for creating change. During the initial phases of the Covid-19 pandemic, it made people accept great interventions in their freedom of movement and daily routines overnight. Arguably, a certain apocalyptic tone is necessary and pandemic narratives need to be dominant in order for pandemic restrictions and measures to be effective. If these narratives were “low mimesis” and “mundane” (Smith, 2005: 20–24) for example, or just one story among many, social distancing would be less effective, and there would be little legitimacy for pandemic measures involving great economic and societal costs. In this sense, apocalyptic stories can play an important role in facilitating favorable change. However, stories with an apocalyptic tone and content, are also particularly fit for arousing criminal and harmful action.

### Master and counter-narratives

Various conceptualizations of dominant stories exist in narrative and discourse theory. Foucault (1970) famously described them as episteme, referring to overarching systems of knowledge that are taken for granted in particular historic periods. In his later studies of sexuality however, he moved away from these grand historical epistemes toward specific discourses struggling for domination in more limited and specific social fields (Foucault, 1978). These discourses bear a resemblance to what other theoretical approaches understand as formula stories (Loseke, 2007), cultural stories (Richardson, 1990) or master narratives (Clifton and Van De Mieroop, 2016). For the purposes of this article, we use the concept of master narratives to describe dominant stories, and understand them as centers of power “directed from and ultimately constructed and reproduced as social reality by dominant institutions, agents and systems” (Snajdr, 2013: 234).

Clifton and Van De Mieroop (2016: 7) argue that master narratives are flexible and may be used for different purposes, and conclude that people tend to be oriented toward master narratives “in one way or other”, by, for example, negotiating with them (Plummer, 2019), or opposing them in counter-narratives (Bamberg and Andrews, 2004). Counter-narratives are “stories that challenge or oppose dominant stories, either in mainstream social or in subcultural contexts” (Sandberg and Colvin, 2020: 4), and these may include components of master narratives as part of their narrative resistance (Andrews, 2002). The main difference between counter- and negotiated narratives is that counter-narratives repudiate and argue with the dominant narrative, while negotiated narratives develop “weapons to resist while not challenging the existing order” (Plummer, 2019: 15).

Counter-narratives have often been understood rather romantically, as the voice of the marginalized, fighting stigmatization, oppression and cultural domination (e.g. Canham and Malose, 2017; Joosse et al., 2015; McKenzie-Mohr and Lafrance, 2017; Sandberg and Colvin, 2020). In fact, however, the countering has no intrinsic moral value, and as we will show below, counter-narratives may well create harm or be “criminogenic”; that is, they may drive, encourage, arouse or motivate crime. In criminological theory, some subcultures (for example the subculture of violence (Wolfgang and Ferracuti, 1967); or street culture (Bourgois, 2003) encompass criminogenic counter-narratives, which promote crime by directly conflicting with, or being in opposition to, the dominant culture. Neutralization theory and neutralization techniques (Sykes and Matza, 1957), on the contrary, can be seen as criminogenic negotiated narratives, as they justify, and thus make it easier to commit crime by appealing to dominant narratives in society.

In this article, we discuss how pandemic master narratives can motivate acts defined as illegal, how these can be negotiated in such a way that they become criminogenic and how some counter-narratives also encourage crime. Together, we show the complex relationship between crime, power and narrative. We also describe how the atmosphere of pandemics creates a narrative environment, or epidemic psychology, that offers opportunities to individuals and groups engaged in criminal activity. It is important to note that describing something as a narrative says nothing about the veracity of the story. A narrative may (or may not) be true, but it still has a particular effect, which is the emphasis of this study.

Heeding the criticism directed at criminology for theorizing from examples and data from the Global North, and calls for a global or southern criminology (e.g. Aas, 2012; Carrington et al., 2016; Fraser, 2013), our case is situated in Latin America, more specifically Mexico, during the early phases of the coronavirus pandemic.

## The coronavirus pandemic in Mexico

The first case of Coronavirus was confirmed in Mexico City on 28 February 2020. A few hours later, another case was reported in Sinaloa and a third, once again, in the capital. The first death from Covid-19 occurred 20 days later. By 20 November 2020, 100,000 deaths made Mexico the number four country in the world in registered Covid-19 deaths (after USA, Brazil and India). Due to limited testing numbers are probably much higher. Approximately a month after the first case was reported, the government implemented a series of measures aimed at preventing and controlling the spread of infection. For example, it extended the Easter school vacations,^1^ and implemented the National Day of Social Distancing^2^ and the DN-III-E Plan (providing aid to the civil population in the event of disaster).^3^ On 24 March, phase 2 of three epidemiological phases (depending on transmission rates) was announced, comprising the suspension of certain economic activities, the prohibition of mass gatherings and the recommendation for the general population to remain at home. On 30 March, a “health emergency” was declared, and on 21 April, the start of phase 3 was announced, which increased and extended restrictions on movement, and prevention and control activities.^4^

After the first coronavirus death in Mexico, the General Health Council classified Covid-19 as a “serious disease of priority attention”.^5^ Shortly after, the Undersecretary of Prevention and Health Promotion Dr López-Gatell Ramírez, began an intense information campaign focusing on personal care to prevent contagion including emphasis on: continuous hand washing, taking care while sneezing, suspension of non-essential activities and self-confinement.^6^ He announced many measures and issued warnings in conferences held by the President López Obrador. At the same time, the President was reluctant to commit fully to the messages from the Undersecretary. He did for example continue traveling, and infamously stated that: “Look, about the coronavirus, this business that we can’t hug. We should hug. Nothing will happen.”^7^ During the first weeks of the pandemic in Mexico, there was thus a striking interdiscursivity (Fairclough, 2003) in the way that the disease was communicated through official channels. The WHO-sponsored pandemic master narrative and a version of a pandemic counter-narrative co-existed, although the one emphasizing the gravity of the disease dominated, and was generally supported by mass media. This ambiguity was also reflected in daily life with crowded street markets without any preventive measures^8^ coexisting with empty streets elsewhere, closures of schools and images of soldiers evicting tourists from the beaches of Acapulco.^9^

Mexico struggles with poverty, great inequalities and a long-lasting and upward spiraling trend in homicides and crime. The State is weak and drug cartels and criminal organizations control parts of the territory. They also take or try to take on the role of the State in certain regions (Durán-Martínez, 2017). The Jalisco Nueva Generación Cartel (CJNG) for example, built a hospital in Jalisco.^10^ During the first months of the coronavirus pandemic, drug cartels similarly tried to challenge and take on the role of federal and state authorities. Mexico is also a fragile democracy having been ruled by one party (PRI) until 2000, corruption is widespread and there is low trust in both federal and state governments. Illustratively, about half of the population doubts the government responses and the health system’s capability to cope with the pandemic.^11^ Reports of infection and death rates have been questioned regarding the possible underreporting of figures (for example, there are discrepancies between numbers reported by individual states and the federal government).^12^

The economic effects of the coronavirus pandemic have made the economic situation worse and arguably increased political polarization and further lowered trust in government institutions. Accordingly, there have been several affective and sometimes ferocious responses to the pandemic, across the country. These vary from protests by medical personnel about the lack of basic protective gear,^13^ to protest against corona measures, looting (particularly in the beginning) and roadblocks. Looting has a certain history in Mexico, for example as a protest against rising prices on fuel or transportation.^14^ Roadblocks are often used in political protests too,^15^ but got a new meaning as a means of limiting the spread of the virus during the coronavirus crisis.

## Storytelling, pandemics and crimes

Our ideas, overview and insight into what we describe as “corona crimes”, developed through a process of web scraping thousands of news articles on crime and corona during the first months of the coronavirus pandemic in Latin America. This article, however, is a theoretical reflection on the early phases of the pandemic, drawing on only a few illustrative examples from Mexico, the context we know best. Our aim is to conceptualize and understand crimes that (may) appear, or are reignited in the wake of pandemics, and how these are connected to early pandemic narratives.

### Crimes driven by pandemic master narratives

During the first months of Covid-19, the disease was often narrated as an overwhelming danger to health and societies’ normal way of life. In response, both governments and the general population undertook beyond-normal efforts to halt its spread. One of the main stories of the coronavirus pandemic is that human mobility spreads the disease and should be restricted. This master narrative is the foundation of many governmental and municipal strategies for fighting the disease, from limitations on travel and social distancing, to school and business closures. Backed by science, the mobility-story can be seen as the most important or influential master narrative of the coronavirus crisis. It works to justify relatively profound interference in people’s lives by governments, but the story also has other consequences.

In Mexico, this master narrative has resulted in incidents where groups of citizens have attempted to illegally close roads or other access points in order to protect their communities, or have otherwise tried to hinder human mobility to avoid contagion. Armed with weapons, local residents have blocked access to hundreds of municipalities across the country.^16^ The town of Tepotztlan, which every week welcomes 20,000 visitors, was “closed” by residents to prevent tourists from entering. “We are checking license plates and licenses. If they are from Mexico City, they cannot pass”, stated those responsible for the roadblock. Armed with signs that read, “It’s not vacation time”, residents positioned themselves at the main entrance to the town and on the “La Pera-Cuatla” highway to prevent access.^17^ In another case, a family that had been to a hospital in Mexico City with their baby daughter for a non-corona-related heart problem, was denied entry to their home by local officials in a town in Guerrero.^18^ Cases in Yucatan have involved laid off or suspended employees of the tourism industry being denied access to their home villages out of fear that they will bring the disease with them.^19^ These are just a few examples of the widespread illegal restrictions on human mobility that took place throughout Mexico during the first months of the pandemic. Following the rationale of minimizing human mobility, and contrary to government and state regulations, people have been denied access to their homes, families and work. Although these acts may differ in many ways, for example how roadblocks are enforced, by whom and how such actions were sanctioned by the authorities, they reflect the shared sentiment that governments were not doing enough to stop the spread of the virus and thus citizens felt obliged to take matters into their own hands.

There has been a fine line between self-protection and vigilantism during the Covid-19 pandemic. In many ways, governments and other official institutions looked to local initiatives to control the disease. They also relied on an apocalyptic pandemic narrative and a certain degree of fear in the population, to justify their own, sometimes harsh, interventions and measures. The actions of individuals, groups and communities who go too far in their attempts to constrain the virus and break the law, can be seen as a result of the affective atmosphere created by official institutions and the media. Crimes, such as roadblocks and the denial of human mobility, can thus be seen as the criminogenic side effects of the pandemic master narrative. Perpetrators are expanding the underlying rationale of government sponsored pandemic master narratives, and are executing them in their own way.

When crimes are driven by official narratives, it may be reflected in the response of the authorities. In many cases it appears as if law enforcement is reluctant to intervene, possibly because it understands that the fear driving these incidents is the same as that used to justify its own regulations and measures. Another important reason that governments might be reluctant to intervene, relates to who the perpetrators are. There are examples in Brazil^20^ and El Salvador^21^ of criminal gangs enforcing curfews and other actions to control the spread of the virus, but the groups behind roadblocks and similar actions in Mexico were not linked to organized crime. These people were not the usual suspects of crime, which may help explain the relatively restrained law enforcement responses. This resonates with a well-known observation in narrative studies (e.g. Fleetwood, 2016; Polletta, 2006): the credibility of storytellers depends not only on the trustworthiness of their story, but also on their pre-established position in society.

### Crimes aroused by negotiated pandemic master narratives

Medical epidemics and pandemics can generate epidemics of fear, suspicion and irrationality—and related stigmatization (Strong, 1990). The sometimes-apocalyptic narratives that dominate, especially in the early phases of a pandemic, create an environment of panic and suspicion where people search for scapegoats and may be willing to consider rather drastic means to counter the disease. The fear triggered by pandemic narratives may for example be the reason for attacks on stigmatized groups. In a state of epidemic psychology this can happen both to “those with the disease and to those who belong to what are feared to be the main carrier groups” (Strong, 1990: 253).

Health workers have been over-represented in statistics of coronavirus infection and death rates all over the world. In Mexico, for example, almost 104,590 health workers were infected and 1410 had died (49% were doctors and 18% were nurses) as of 3 September 2020 (the highest number in the world at that time).^22^ Health workers tend to pay particular attention to safety measures in order to not catch the disease, and sometimes leave their families and live alone so as to avoid spreading it. Their considerable personal sacrifices have therefore often earned them the role of the main heroes (Propp, 1968) in pandemic master narratives. In a negotiation (Plummer, 2019) of these pandemic master narratives, however, it has also been argued that given that health workers are especially at risk of contracting the virus, their mobility and contact with others should be particularly constrained. This negotiated version of the pandemic master narrative has motivated attacks on health workers and hospitals all over Latin America.

In Mexico, 21 such attacks had been recorded by April 2020,^23^ although there were probably many more. In Yucatán, a man threw a cup of hot coffee over a nurse as she left a supermarket, shouting “you are infecting us all!”^24^ In Queretaro, two women were arrested for attacking a health worker on the street after accusing her of having Covid-19,^25^ and in Oaxaca a doctor was attacked with bleach because he had treated coronavirus patients. The attacker claimed that he would “disinfect him”.^26^ In Guerrero, Red Cross workers have also been beaten and attacked with bleach, allegedly to remove the virus.^27^ Another version of these attacks on health workers has occurred at hospitals, where people have gathered to apparently prevent the spread of the virus. In Morelos, around 150 people congregated outside a hospital to demand that no Covid-19 patients be admitted, and threatened to burn down the hospital if they were.^28^ It was feared these patients could infect others and that the hospital would not have the capacity to treat them.

Attacks on health workers are motivated by fear of infection and the spread of the disease in the street, public transportation, supermarkets as well as local hospitals. Sometimes they can take on a highly symbolical meaning, as when victims are attacked with bleach, and health workers are considered “matter out of place” in public spaces (Douglas, 2009: 44). These attacks can be seen as encouraged by a negotiated pandemic master narrative, where one of the main narrative characters (Propp, 1968), are converted from heroes fighting the disease to villains spreading it. Arguably, the fear of infection from high-risk groups, and demands that they should not use public transport, enter stores or be on the street, in fact follow the logic of the pandemic master narratives which state that groups who are particularly at risk of infection should be quarantined and self-isolate as much as possible. These negotiated criminogenic narratives demonstrate how closely intertwined master and counter-narratives can be (Bamberg and Andrews, 2004). Identifying, partly stigmatizing and limiting the mobility of high-risk groups is in accordance with the pandemic master narrative. However, the way this is done, (through violent attacks), and especially to whom (those combatting the disease), is vastly different.

As the victims of these attacks are the heroes of the pandemic master narrative, the attacks are particularly problematic in the public eye. Sanctions are therefore often severe. Even in a country like Mexico, with high levels of impunity, and where few crimes are investigated, the police did investigate and arrest all those involved in the attacks on medical personnel, including those who had “just” insulted nurses.^29^ The Mexican Senate even launched an initiative to increase prison terms and economic sanctions for aggressors of healthcare workers (eight years’ effective prison).^30^ The health worker as hero is an important story for governments and officials. It motivates these workers for the important job they do and foments public acknowledgment.

### Crimes driven by pandemic counter-narratives

Foucault (1978: 95–96) famously claimed that where “there is power, there is resistance, and yet, or rather consequently, this resistance is never in a position of exteriority in relation to power”. This connects to the topic of this study, as crimes and other societal harms can also be driven by counter-narratives to pandemic master narratives. Counter-narratives challenge or oppose dominant stories in a multitude of ways (e.g. Bamberg and Andrews, 2004; Canham and Malose, 2017). While negotiated narratives may sometimes be considered counter-narratives, in this section of the analysis, we refer to more clear-cut counter-narratives that dismiss pandemic master narratives by rejecting their central truth claims.

The most significant counter-narrative to Covid-19 pandemic master narratives describes the pandemic as a conspiracy driven by people in power, and emphasizes that it is being used as a means of governmental control. As all narratives, it comes in different versions, the most extreme being that coronavirus does not in fact exist, to others that claim that it is nothing more than the “regular flu” being used opportunistically by the system. The law breaking that follows such counter-narrative is usually breaches of government regulations such as contravening government, state or municipal curfews or business closure regulations, but these counter-narratives can also arouse violent demonstrations and vandalism.

There have been widespread violations of government regulations throughout Latin America. In many countries, public areas such as beaches have been closed off, and in Mexico, police have been heavy-handed in enforcing these new laws.^31^ In Mexico City, where coronavirus measures shut down non-essential businesses for months, shop owners found creative ways to continue to do business. Some had employees in the street whispering to passers-by and letting customers in through apparently closed doors,^32^ and a bicycle manufacturer tried to hide 350 employees when officials came to inspect his premises.^33^ In another case in Jalisco, a gym and steam bath, located in front of the Atemajac light rail station, continued to operate despite their supposed closure and being considered a hotspot of infection (clients arrived individually and their entry was authorized through a side door).^34^ Similarly, in Tabasco, along the city’s Periferico and Ruiz Cortines avenues, stores selling construction material, mechanics and motorcycle workshops, among others, continued to operate normally.^35^

While it is true that most of these incidents were probably motivated by self-interest or perhaps just negligence, they are all made easier, and perhaps more common, by the underlying counter-narrative that the coronavirus crisis has been exaggerated by powerful groups in society. In a classic dilemma of the “tragedy of the commons” it is in most people’s interest for everyone else to follow restrictions while they do not. There is also a distinct class-dimension involved in these breaches of government regulations; “those with gardens and cars are better placed to obey them than residents of densely populated cities” (Fleetwood et al., 2020).

On other occasions, however, support of counter-narrative has been more explicit. In Chiapas, a hostile crowd attacked a hospital claiming that coronavirus was “a lie” and “a plot to kill people” and tried to release patients.^36^ In the same state an angry mob went on a rampage, shouting that “the coronavirus doesn’t exist”,^37^ and in Yucatan, a group of people attacked a police station and held the chief of police hostage until he promised to reopen parks and public spaces.^38^ The pandemic counter-narrative has also garnered support from people in power, adding a layer of complexity and nuance to the relationship between official master narratives and counter-narratives. The Mexican President, for example, was hesitant to support the World Health Organization (WHO) and Mexican health authorities’ pandemic narratives during the first months of the disease. He continued traveling, encouraged people to go out shopping, hugged his supporters and avoided the use of face masks. After a while however, he fell more into line with the master narratives, although was still more half-hearted about it than other representatives of the government and state apparatus. Some philosophers, economists and other representatives for the academic elite have also objected to government regulations, as seen, for example, in the Agamben controversy.^39^ In general, however, pandemic master narratives have been fronted by officials while counter-narratives have emerged “from below”.

Depending upon the framing, violation of government measures, attacks on hospitals and police stations and so on, can be seen as anything from opportunistic criminal behavior to resistance to an oppressive and authoritarian political regime. If policies are presented as unnecessary, strict or undemocratic, breaking them becomes an act of defiance against undemocratic or illegitimate governments. The famous phrase “one man’s terrorist is another man’s freedom fighter” encapsulates this well. These narrative struggles are particularly important within the Latin American context where it can be argued that some governments, such as in El Salvador, Guatemala and Columbia have taken political advantage of the crisis to control the population and opposition, and to justify human rights violations (e.g. Sanchez Parra, 2020).^40^ Even in Mexico, which has been more lenient in implementing corona measures, a man was beaten to death by police in Jalisco, for not wearing a face mask.^41^ This sparked nationwide demonstrations that were violently suppressed in many places.^42^ The pandemic counter-narratives are, in this sense, not criminogenic nor “wrong” per se, but raise questions about the moral and political authority of the master narratives and the dominant institutions, agents and systems that promote them. Counter-narratives and the law breaking that follows them thus, rightly or wrongly, challenge societal agreements and State definitions of what should be understood as crime.

### Changing the meaning of “crime” and “criminals” via pandemic narratives

Societal crises and catastrophes such as pandemics not only inspire new forms of violation of the law, but also reignite and change the meaning of old ones. For “a moment at least, the world may be turned upside down” (Strong, 1990: 255), opening up for “moral entrepreneurs” (Becker, 1963). Pandemic master narratives offer an opportunity for justification, for example through neutralization techniques such as “denial of responsibility” or “appeal to higher loyalties” (Sykes and Matza, 1957). They may also provide opportunities for people to craft new stories of themselves, their communities and organizations. Throughout history, wars for example, have mobilized “criminals” who have seized the opportunity to modify or change their social position (see, for example, Basra and Neumann, 2016).

During the pandemic in Mexico, there were several cases of looting in Guerrero, the State of Mexico, Mexico City, Oaxaca, Yucatan and Guanajuato, all justified by pandemic narratives. Groups used social media to call for the raiding of stores and supermarkets, sometimes even going so far as to specifically name stores, locations and types of goods to be stolen. Fifty percent of the 89 incidents of attempted looting during the Covid-19 pandemic were concentrated in five states: the State of Mexico had 16 cases; Mexico City registered 14; Nuevo Leon had 7; and Veracruz and Baja California had four each. Calls for looting also occurred in 17 other states.^43^ By as early as late March 2020, with only a few reported cases of coronavirus, authorities in the State of Mexico had identified 45 Facebook profiles that promoted looting. Most of these had been involved in previous calls for looting under various pretexts, and this time the Covid-19 prevention measures were used to justify citizens’ right to loot.^44^

In Mexico, Facebook pages which had previously been used to call for looting were once again used during the coronavirus crisis. This indicates that the motivation for looting was less directly connected to the health crisis and more a pretext for opportunistic behavior. Most other cases of looting in Mexico, however, were less clear-cut. In times of crisis, it can be difficult to distinguish between looting and other crimes that occur out of necessity, as well as to identify offenders who may use the pandemic as a justification for crimes they would have committed anyway (or would have wanted to). Times of crisis thus frequently intensify the narrative negotiations of what should be considered a crime. They also offer an opportunity for people to engage in and rationalize behavior considered illegal, immoral or simply wrong (e.g. raping, looting and violence in times of war). The apocalyptic pandemic master narrative is a powerful narrative resource that can be used to justify many behaviors that would otherwise be deemed illegal. When the story behind breaking the law changes, the “crime” also changes, and it is potentially perceived as something else.

The pandemic master narrative can also change popular stories of those who violate the law. In response to the coronavirus crisis, drug cartels distributed food and other goods in several states in Mexico. Armed and openly displaying their criminal affiliations, they were often welcomed as heroes by the people receiving the handouts.^45^ In Guadalajara for example, cartels began to distribute boxes (containing rice, beans, sugar, cookies, pasta, atole—maize puree—different flavored cornstarch, oil and toilet paper) adorned with the image of Joaquin Guzman Loaera, alias “el Chapo”.^46^ This practice was so widespread, that the press began to refer to “narco-solidarity” and “narco-groceries”.^47^ Almost all the known cartels distributed food: in San Luis Potosi, the Jalisco Nueva Generación Cartel distributed food parcels with labels that read “From your CJNG friends, support for the Covid-19 emergency”.^48^

These acts can be seen as an attempt to take over the role of the State and benevolent organizations. They can also be seen as attempts to change dominant stories in a society. Crises, catastrophes and great societal changes, such as pandemics, can alter the fundamental meaning of well-established categories, and thus challenge widely shared meanings of crime and criminal actors. Cartels and criminal gangs can, for example, take advantage of the opportunity provided by the coronavirus crisis, to redefine their role, and shift from villains to heroes in master narratives. The turmoil, unusual circumstances and atmosphere created by the at-times apocalyptic pandemic narratives, can create a narrative openness (Punday, 2012) for such new stories. In this way, societal crises and the stories that accompany them create structural and narrative opportunities, not only for politicians and the powerful elites, who might use them to implement policies to suit their own interests, but also for criminal entrepreneurs on the margins of society.

## Discussion and conclusion

The coronavirus pandemic in Mexico and Latin America has aroused crimes, such as hate or fear crimes against health workers and hospitals, the illegal denial of public mobility out of fear of infection, and also justified looting and other traditional crimes. Violations of pandemic regulations are a new type of crime emerging during the pandemic. There are many ways to approach a study of crimes driven by pandemics, but we argue that it is difficult, if not impossible, to understand these without seeing how they are fundamentally embedded in, and emerge from, pandemic master narratives. Using a narrative criminological approach (Fleetwood et al., 2019), we have highlighted the storied dimension of these “corona crimes”. We have identified the emergence of new crimes, the negotiation and reigniting of more traditional crimes and the opportunities for carving out new roles in a narrative environment dominated by epidemic psychology (Strong, 1990) and apocalyptic pandemic master narratives.

Smith (2005) argues that in apocalyptic narratives the stakes are so high and evil so horrific that room for action becomes extraordinary and cultural constraints for violence and other dramatic actions can be overcome. Apocalyptic pandemic narratives differ from those of, for example, war (Smith, 2005), religious military uprisings (McCants, 2015), environmental rebellions (Joosse, 2019) and pro-life movements (Mason, 2002) in that it is more complicated to identify the enemy. Enemies in apocalyptic narratives share characteristics of the “folk devils” in moral panics (Cohen, 1972), and are associated with anything “profane, polluted, evil and dangerous” (Smith, 2005: 15). However, when the enemy is a virus, it will be necessary, with intense narrative interpretation and negotiation, to identify more “human-like” antagonists. These can be anything from those infected with the disease, or suspected of being infected, to politicians or others in power who have not acted adequately during the crisis. The enemies can thus change from day to day (young partygoers, particular immigrant groups, etc.) following the spread of the disease in particular segments and groups in society. Similar to apocalyptic narratives in natural catastrophes, pandemics generate more narrative work to figure out who to blame, and victims of attacks under the pretext of pandemic narratives can therefore sometimes be unanticipated (e.g. health workers).

Apocalyptic pandemic narratives and destructive pandemic counter-narratives will have particular resonance in polarized, or fragile or failed states, such as in Latin America. When the population does not trust that the government has the capacity or willingness to handle the threat, or suspects it of conspiracy, they may more easily take issues into their own hands (e.g. roadblocks and violent attacks) or break government regulations. While some of these “criminal” outcomes can be seen as new, most importantly violations of pandemic regulations, others have pre-pandemic parallels and histories. For decades, drugs cartels and gangs have tried to take the role of official institutions in weak states in Latin America, to increase their support and legitimacy (Bunker and Sullivan, 2010; Campbell, 2014). The pandemic just offered them another opportunity. Hate against hospitals and health workers aroused by apocalyptic narratives is nothing new either, but has mainly been a US phenomenon, related to abortion clinics (Mason, 2002). Roadblocks also have a long history in Latin American political protests, but have acquired new meaning as a means of limiting infections (Almeida, 2007; Alvarez et al., 2017; Madrid, 2005). As always there is continuity in change, and new criminogenic stories are based, and further develop, old ones.

Insights into these alterations in the criminal landscape offered by epidemic psychology include how medical pandemics can be followed by epidemics of fear, panic, suspicion and stigmatization, and how this is often particularly concentrated early on in a pandemic. According to Strong (1990: 258): epidemic psychology can “only be conquered when new routines and assumptions which deal directly with the epidemic are firmly in place”. Insights offered by narrative criminology (Presser and Sandberg, 2015) include a fundamental understanding of how the storied framing of law breaking influences, if not determines, how these crimes are understood and sanctioned, and whether they are even considered “crimes” at all. Looting during a pandemic can, for example, be interpreted as an unscrupulous crime or as necessary self-providing; health workers can be seen as heroes fighting the disease or potential carriers of the virus; government regulations may be understood as necessary to avoid a catastrophe or as tools used by an illegitimate regime to control the population; and breaches of government regulations may be anything from selfish risk-taking potentially spreading the disease, to resistance against authoritarian law enforcement and undemocratic governments.

How violations of the law are understood and sanctioned depends on already established perceptions of “criminals” and victims. If the victim is an ideal victim (Christie, 1986) or some kind of hero (Propp, 1968), sanctions are likely to be harsher, while if the “criminal” is perceived as a law-abiding citizen, both the police and the public are likely to be more lenient. Already established socio-economic and cultural structures are always brought into the crime story, influencing both its production and interpretation (Fleetwood, 2014, 2016). However, the interpretative processes on the meaning of crime intensify during societal crisis. Pandemics and other catastrophes thus offer a unique opportunity to change these social structures and establish new patterns of cultural interpretation. The crimes of pandemics can be aroused and legitimated by officially promoted master narratives, but also by negotiated narratives and counter-narratives challenging dominant stories. This study thus demonstrates that crime is a fluid concept, and the result of intense narrative negotiation and creativity by actors with invested interests and institutional positions. Rather than being a clear-cut category or behavior, crime is a narrative accomplishment, and hence the importance of narrative analysis and theory, and narrative criminology when studying it.

The main part of the analysis and discussion is limited to that which is defined as crime by the State, leaving out state crime and other forms of harm that could have been described as “criminal”. Our argument about the social construction of crime could (and arguably should) have been taken even further, emphasizing how harm committed by the State during the coronavirus pandemic should be seen as state crime and not be separated from non-state criminality (e.g. Ross and Barak, 2000). This includes failure to act on early warning, providing essential personal protective equipment, protecting vulnerable groups, securing sufficient testing and generally prioritizing the economy over health. Deaths following such policies can be conceptualized as “forms of violence and even as crimes” (Fleetwood et al., 2020). Moreover, in Baja California, Mexico, the decision to reopen business was described as “criminal” by members of labor unions and social activists.^49^ In this sense, pandemic narratives and their effects are extremely wide ranging in terms of what can be seen as crime, and what is referred to as crime by the public.

Our study is limited in time, geography and prevalence as some of the crimes we describe are relatively rare. Nevertheless, they can still forewarn types of crime and criminogenic narrative interpretations that may occur, or that have already occurred, during other similar crises. Breaches of government regulations are widespread globally, and health workers have been attacked in the USA, Philippines and India.^50^ The counter-narratives to the pandemic master narrative are also not particular to Mexico, nor to Latin America, but may even be stronger in the USA, and have inspired sometimes illegal demonstrations both there^51^ as well as in Germany^52^ and Australia.^53^ Pandemic conspiracy theories have also gone global resulting in violence and vandalism.^54^ The pandemic has also been used globally by governments to contain protest and control the population, for example in Hong Kong (Ismangil and Lee, 2020) and the Philippines (Joaquin and Biana, 2020).

This study addresses the narrative criminological question of how crimes can be seen as maintained, instigated (Presser and Sandberg, 2015) or aroused (Presser, 2018) by narratives. Our theoretical contribution to this framework is highlighting the role of state-sponsored narratives, and specifying how crimes can emerge from such master narratives and the counter-narratives they inspire. This links narrative criminology with studies of the relationship between power and language in other disciplines. We have also revealed how pandemic narratives can be negotiated (Plummer, 2019) in ways that make them criminogenic.

Arguably, the narrative and criminogenic processes we describe may be even more important in future, and possibly deadlier, pandemics. The processes we describe in this article will probably intensify then. Understanding how crimes of pandemics are driven, embedded and negotiated in and by stories, can be helpful when implementing pandemic policies and measures, and when considering how these should be communicated to the public. Studying “corona crimes” can help to avoid unwanted consequences of official pandemic narratives, to identify criminogenic counter- and negotiated narratives, and to identify both state and criminal entrepreneurs’ attempts to take advantage of the narrative openness that follows in the wake of national and international crises.

## Notes

1. https://www.animalpolitico.com/2020/03/sep-adelanta-vacaciones-semana-santa-30-dias-coronavirus/.

2. https://www.gob.mx/cms/uploads/attachment/file/541687/Jornada_Nacional_de_Sana_Distancia.pdf.

3. https://www.gob.mx/sedena/acciones-y-programas/que-es-el-plan-dn-iii-e.

4. https://www.gob.mx/salud/prensa/consejo-de-salubridad-general-declara-emergencia-sanitaria-nacional-a-epidemia-por-coronavirus-covid-19-239301.

5. https://www.infobae.com/america/mexico/2020/04/28/dos-meses-con-la-epidemia-la-ruta-del-covid-19-en-mexico/.

6. https://www.unotv.com/noticias/portal/nacional/detalle/coronavirus-en-mexico-salud-recomendaciones-lavado-mexico-288252/.

7. https://www.eluniversal.com.mx/nacion/del-hay-que-abrazarse-no-pasa-nada-como-anillo-al-dedo-las-frases-de-amlo-sobre-el-covid-19.

8. https://www.france24.com/es/20200329-peticion-amlo-aumento-casos-mexicanos-siguen-calles.

9. https://www.eluniversal.com.mx/estados/policias-y-militares-desalojan-turistas-en-playas-de-acapulco.

10. https://www.infobae.com/america/mexico/2020/07/31/narco-construcciones-hospitales-escuelas-clubes-y-toda-una-infraestructura-al-servicio-del-crimen/.

11. https://www.eleconomista.com.mx/arteseideas/Covid-19-mexicanos-entre-preocupados-y-desconfiados-20200302-0147.html.

12. https://datos.nexos.com.mx/?p=1388.

13. https://www.eluniversal.com.mx/nacion/medicos-protestan-por-falta-de-insumos.

14. https://www.infobae.com/america/mexico/2018/01/06/preocupacion-en-mexico-por-los-saqueos-organizados-desde-las-redes-sociales/.

15. https://traficozmg.com/2019/07/bloquearan-carreteras-en-todo-mexico-por-protestas/.

16. https://mexiconewsdaily.com/news/coronavirus/municipalities-dont-have-authority-to-restrict-access-feds/.

17. https://www.elfinanciero.com.mx/estados/municipios-turisticos-en-morelos-cierran-acceso-a-visitantes-para-contener-contagios-de-covid-19.

18. https://mexiconewsdaily.com/news/coronavirus/guerrero-family-barred-from-returning-home-for-fear-of-contagion/.

19. https://mexiconewsdaily.com/opinion/virus-threat-leaves-yucatan-tempers-rising-with-the-temperatures/.

20. https://www.theguardian.com/world/2020/mar/25/brazil-rio-gangs-coronavirus.

21. https://www.latimes.com/world-nation/story/2020-04-07/el-salvador-coronavirus-homicides-bukele.

22. https://www.forbes.com.mx/noticias-mexico-pais-con-mas-personal-medico-muerto-por-coronavirus/.

23. https://www.economist.com/international/2020/05/11/health-workers-become-unexpected-targets-during-covid-19.

24. https://mexiconewsdaily.com/news/coronavirus/nurse-attacked-with-hot-coffee/.

25. https://mexiconewsdaily.com/news/coronavirus/2-women-arrested-in-queretaro-for-attacking-health-worker/.

26. https://mexiconewsdaily.com/news/coronavirus/doctor-attacked-in-oaxaca-nurse-evicted-in-sonora/.

27. https://mexiconewsdaily.com/news/coronavirus/volunteer-paramedic-beaten-attacked-with-bleach/.

28. https://mexiconewsdaily.com/news/coronavirus/residents-threaten-to-burn-hospital-if-covid-19-patients-treated/.

29. https://www.infobae.com/america/mexico/2020/05/19/ingresaron-a-prision-a-un-hombre-de-queretaro-que-agredio-a-su-vecina-por-ser-enfermera/.

30. https://heraldodemexico.com.mx/pais/partido-verde-propone-penas-de-prision-para-atacantes-de-medicos-enfermeras-y-personal-que-atiende-covid-19/.

31.
https://mexiconewsdaily.com/news/coronavirus/cops-get-heavy-handed-over-face-mask/
https://mexiconewsdaily.com/news/investigation-of-puerto-vallarta-police-urged-for-alleged-abuse/.

32. https://mexiconewsdaily.com/news/coronavirus/shop-owners-attempt-to-avoid-coronavirus-closure-order/.

33. https://mexiconewsdaily.com/news/coronavirus/bicycle-maker-attempted-to-hide-350-employees-from-inspectors/.

34. https://www.informador.mx/jalisco/Establecimientos-burlan-cierre-pese-a-ser-foco-rojo-de-contagios-20200525-0007.html.

35. https://www.tabascohoy.com/burlan-decreto-siguen-abiertos/.

36. https://mexiconewsdaily.com/news/coronavirus/mob-attempts-to-rescue-patient-from-chiapas-hospital/.

37. https://mexiconewsdaily.com/news/coronavirus/citizens-go-on-rampage-in-chiapas/.

38. https://mexiconewsdaily.com/news/facilities-in-yucatan-town-reopened-then-closed/.

39. See the full debate in the *European Journal of Psychoanalysis*: http://www.journal-psychoanalysis.eu/coronavirus-and-philosophers/.

40. https://www.bbc.com/mundo/noticias-america-latina-52501875.

41. https://mexiconewsdaily.com/news/police-accused-in-death-of-man-arrested-for-not-wearing-face-mask/.

42. https://www.eluniversal.com.mx/english/giovanni-lopez-mexican-protesters-clash-jalisco-police-over-police-brutality-death.

43. https://www.animalpolitico.com/2020/05/estados-intentos-saqueo-epidemia-covid-19/.

44. https://mexiconewsdaily.com/news/pandemic-used-as-pretext-to-promote-looting-on-social-media/.

45. https://mexiconewsdaily.com/news/cartel-continues-distributing-supplies/.

46. https://www.elsaltodiario.com/coronavirus/narcosolidaridad-en-mexico-los-carteles-aprovechan-el-coronavirus-para-disfrazarse-de-ong.

47. https://www.infobae.com/america/mexico/2020/04/25/el-dia-despues-como-cambiara-el-coronavirus-al-narco-en-mexico/.

48. https://www.infobae.com/america/mexico/2020/04/20/narcos-aprovechan-coronavirus-en-mexico-para-repartir-despensas-y-pelear-territorio/.

49. https://mexiconewsdaily.com/news/coronavirus/baja-california-makes-criminal-decision-to-reopen-100-businesses/.

50. https://www.economist.com/international/2020/05/11/health-workers-become-unexpected-targets-during-covid-19.

51. https://www.washingtonpost.com/nation/2020/05/13/protest-violence-coronavirus/.

52. https://www.tagesschau.de/inland/corona-proteste-109.html.

53. https://www.sbs.com.au/news/conspiracy-theorists-flout-social-distancing-rules-at-melbourne-coronavirus-lockdown-rallyhttps://www.theguardian.com/world/live/2020/may/10/australia-coronavirus-live-updates-nsw-victoria-qld-restrictions-latest-news-update.

54. E.g. https://www.politico.eu/article/coronavirus-5g-conspiracy-mast-burning/.
